# Determinants of tetanus immunization among pregnant women where tetanus has not been eliminated: a multilevel analysis of 6 countries

**DOI:** 10.3389/fgwh.2025.1481771

**Published:** 2025-03-13

**Authors:** Werkneh Melkie Tilahun, Habtamu Geremew, Lamrot Yohannes Abay, Adugnaw Bantie Kebie, Mulat Belay Simegn

**Affiliations:** ^1^ Department of Public Health, College of Medicine and Health Sciences, Debre Markos University, Debre Markos, Ethiopia; ^2^ College of Health Science, Oda Bultum University, Chiro, Ethiopia; ^3^ Department of Environmental and Occupational Health and Safety, College of Medicine and Health Sciences, Institute of Public Health, University of Gondar, Gondar, Ethiopia; ^4^ Department of Pediatrics and Child Health Nursing, College of Medicine and Health Sciences Debre Markos University, Debre Markos, Ethiopia

**Keywords:** tetanus immunization, pregnant women, maternal and neonatal tetanus elimination, determinants, multilevel analysis

## Abstract

**Background:**

Two or more doses of the tetanus toxoid (TT) vaccine in pregnancy afford the fetus passive immunity and reduce neonatal mortality by 96%. In developing nations, the use of TT during pregnancy is still uncommon but presents a serious risk to public health. Thus, the current study aimed to identify determinants of adequate TT immunization among pregnant women in six countries that have not eliminated maternal and neonatal tetanus.

**Methods:**

A cross-sectional study was conducted using recent demographic and health survey datasets from 6 countries that didn't achieve maternal and neonatal tetanus elimination. A total of 84,248 weighted samples were included. A multilevel logistic regression model was fitted. An adjusted odds ratio with a 95% CI and *p*-value < 0.05 was used to declare significant factors.

**Results:**

Being married [AOR = 1.36, CI: 1.20, 1.54], poorest [AOR = 1.46, CI: 1.36, 1.57], the poorer [AOR = 1.48, CI: 1.39, 1,59], middle [AOR = 1.33, CI: 1.26, 1.42], and the richer [AOR = 1.19, CI: 1.13, 1.26] wealth quintile, giving birth between the ages of 24 and 30 years [AOR = 1.10, CI: 1.04, 1.16], being primiparous [AOR = 1.09; CI: 1.02, 1.17], female house head [AOR = 1.13; CI: 1.06, 1.20], 4 and above antenatal care (ANC) visits [AOR = 5.94, CI: 5.60, 6.30], attending post-natal checkup [AOR = 1.18, CI: 1.13, 1.23], and institutional delivery [AOR = 1.22, CI: 1.18, 1.27] were positively related to adequate TT immunization. While unemployment [AOR = 0.68, CI: 0.66, 0.71], poor health facility visits [AOR = 0.72, CI: 0.70, 0.75], abortion [AOR = 0.89, CI: 0.85, 0.93], low community media exposure [AOR = 0.74, CI: 0.67, 0.81], and rural residence [AOR = 0.80, CI: 0.77, 0.84] were significant risk factors for inadequate TT immunization.

**Conclusion:**

Marital status, wealth index, age at first birth, decision about women's health care, parity, sex of household head, ANC, postnatal checkup, distance to health facility, and health insurance were significant predictors of adequate TT vaccination. Therefore, TT immunization can be improved by promoting maternal employment, improving post-abortion care, media coverage, community literacy, and health accessibility, and encouraging pregnant women to receive ANC and postnatal care.

## Introduction

Tetanus is a fatal and contagious infection that is brought on by the spore-forming bacteria Clostridium tetani. It is common in areas of poor hygiene, most disadvantaged, economically poor, and without access to adequate health services (1).

Even though people of all ages can get tetanus, pregnant women have a higher chance of getting the fatal disease (1, 2). Maternal tetanus can occur during pregnancy or within six weeks of termination of pregnancy (3–5) and is responsible for 5% and 14% of all maternal and neonatal deaths worldwide, respectively (6). Around 15,000–30,000 annual maternal deaths occurred due to inadequate tetanus toxoid (TT) vaccination (7, 8).

The use of the TT vaccine is one of the most effective preventative measures and has long been established in many countries (9). The World Health Organization (WHO) has provided guidelines for the immunization of pregnant women against tetanus since 2006 (9–12). A pregnant woman is required to receive three doses of the vaccine to protect her and her newborn(s) from tetanus (13). It can also afford the fetus passive immunity and reduce neonatal tetanus mortality by 96% (14, 15).

Currently, two and above TT doses (TT2+) immunization coverage among pregnant mothers accounts for 75% worldwide, ranging from 95% in Southeast Asia to 53% in the East Mediterranean and 63% in Africa (16). Neonatal tetanus was to be eradicated in 59 priority nations, according to a 1989 World Health Assembly declaration. Currently, only 10 countries (Afghanistan, Angola, the Central African Republic, Nigeria, Pakistan, Papua New Guinea, Somalia, Sudan, South Sudan, and Yemen) remain to achieve maternal and neonatal tetanus elimination (MNTE) (17). Despite significant advancements in the past two decades (18), the use of TT among pregnant women still remains low and presents a significant public health risk, particularly in developing countries in Asia and Africa (4, 19, 20).

Previous studies indicated that factors such as maternal age (21–24), educational status (5, 21, 23, 25, 26), wealth index (5, 21, 24, 26–29), marital status (22, 30), employment (27, 29), media exposure (21, 25, 27), number of ANC visits (21–23, 25–27, 29, 30), place of delivery (5, 21), parity (31), iron uptake during pregnancy (27), decision on women's health care (29, 32), health facility visits (24, 28), health insurance (24), postnatal checkup (33), age at first birth (21, 34), residence (5, 27, 31, 35), distance from health facility (21, 25, 28, 31, 36), and community illiteracy (27) were related to TT vaccination.

If we are to lessen the impact of MNT, we must comprehend the factors that affect pregnant women's decisions to obtain TT vaccinations. This is crucial in countries where MNT hasn't been fully eradicated. The factors that lead to pregnant women receiving an adequate dose of the TT vaccine have not been thoroughly studied. Our study is important in order to help with the process of eliminating maternal and neonatal tetanus.

## Objectives

The goal of this study was to identify factors that contribute to sufficient TT immunization among pregnant women from nations that have not eradicated tetanus.

## Methods and materials

### Data source, sampling and population

This cross-sectional study was conducted based on recent demographic and health survey (DHS) datasets from six countries [Afghanistan (2015), Angola (2015/16), Nigeria (2018), Pakistan (2017/18), Papua New Guinea (2016–18), and Yemen (2013)] that didn't achieve MNTE by 2024 (17). These surveys are nationally representative household surveys, which are conducted at intervals of 5 years in every country and provide a wide range of data on health and related characteristics like population and nutrition. Its samples were typically chosen using a two-stage, stratified cluster sampling method. Each country was divided into enumeration areas, clusters, or communities. By dividing each cluster into urban and rural regions, stratification was carried out. Thereafter, a sample of households was selected from the chosen urban and rural enumeration areas. Data was extracted from (http://www.dhsprogram.com) based on an official online request and permission. The DHS has datasets for different populations, such as women and households (37).

Our study was conducted based on individual record (IR) files. In these datasets, reproductive-age women were included. In our study, 84,248 weighted samples of reproductive-aged women who had births within the five years preceding the surveys were included. Regarding missing values and survey datasets included, of the 10 countries that didn't achieve MNTE, we included 6 countries. This is due to the lack of accessible survey datasets in Somalia and South Sudan; the outcome variable in the Sudan dataset was not recorded, and the survey dataset from the Central Republic of Africa omitted many important variables. The DHS guideline was used to manage any missing data in the dataset.

### Variables of the study

#### Outcome variable

The outcome variable in our study was whether a woman received sufficient TT immunizations during her last pregnancy. The WHO guidelines for appropriate interval for protection at birth defined sufficient TT immunization as receiving two or more doses during current pregnancy; or one dose during current pregnancy and at least one dose before current pregnancy; or at least two TT doses before the current pregnancy, of which the last dose was <3 years before the birth; or three doses within 5 years of the current pregnancy; or four doses with the last dose <10 years before the pregnancy or receiving five doses or more before the current pregnancy (27, 38). Thus, pregnant women who took two or more doses of the TT vaccine during their last birth were considered adequately immunized.

#### Independent variables

We categorized the explanatory variables of this study into individual and community-level factors. Individual-level factors included maternal age, sex of the household head, marital status, women's media exposure, maternal occupation, wealth index, parity, decision on women's health care, health visits in the last 12 months, number of ANC visits, iron supplementation, health insurance, post-natal checkup, and history of termination of pregnancy. Residence, distance to health facilities, place of delivery, community media exposure, and community poverty were considered community-level factors.

#### Measurements and operational definitions

See Table 1.

**Table 1:** Measurement and operational definitions of variables.

Individual level factors	
Age of the mothers	It has been reclassified as “15–24,” “25–34,” and “35+” years to represent the age of women now.
Marital status	The classification of the mother's marital status is “married,” “single,” “divorced,” “widowed,” or “separated.”
Sex of the household head	Taken as it is categorized in the DHS datasets (“male” or “female”)
Women media exposure	Created by combining whether a respondent reads a newspaper or magazine, listens to the radio, or watches television and coded as “yes” (if a woman had history of exposure to at least one of these media) and “no” otherwise.
Maternal occupation	It is the current working status of the respondents and is taken as categorized in the DHS datasets.
Health care visits	It is health care visits in the last 12 months, taken as categorized in the DHS datasets (“yes” or “no”).
Parity	It is the number of times that a woman has given birth to a fetus with a gestational age of 24 weeks or more, regardless of whether the child was born alive or was stillborn, and categorized as primipara (one birth), multipara (2–4 births), and grand multipara (5 or more births) (39).
Decision on women's health care	It is the person who usually decides on women's health care and is categorized as “respondent alone”, “joint decision”, “husband alone”, and “other”.
Number of ANC visit	It is the number of antenatal visits a woman had and is categorized as “no”, “1–3”, “≥ 4” and “don't know”.
Iron supplementation	It indicates whether women were given/bought iron tablets/syrup during pregnancy and is taken as categorized in the DHS datasets.
Postnatal check up	It indicates whether a woman gets a postnatal checkup for her child within 2 months and taken as categorized in the DHS datasets
Ever had terminated pregnancy	It indicates whether a woman had a history of any termination of pregnancy and coded as “no” and “yes”
Household level factors	
Wealth index	It is the household wealth status and is taken as categorized in the DHS datasets. Which includes poorest, poorer, middle, richer, and richest wealth quantiles.
Health insurance	It indicates whether the health care costs are covered by insurance or not and is taken as categorized in the DHS datasets
Distance to health facility	The distance from the healthcare facilities determines whether it is considered a “big problem” or “not a big problem.”
Community level factors	
Residence	The mother's residence is classified as “urban” and “rural.”
Place of delivery	It was defined as at “home and other” and “health facility”.
Community-level media exposure	Aggregated variable from women media exposure and measured by proportion and categorized as low (communities with <50% of women exposed) and high (communities with ≥50% of women exposed).
Community-level poverty	Aggregated variable from household wealth status (proportion of women from the poorest and poorer quantiles) and categorized as low (communities with <50% of women are poor) and high (communities with ≥50% of women poor).

#### Statistical analysis and model building

We merged datasets from six (6) countries. Data extraction, appending, re-coding, and statistical analysis were performed using STATA version 16. Descriptive statistics were analyzed and presented as frequency, percentage, text, and tables. In order to adjust for under-reporting and over-reporting in the surveys, a weighting factor (v005/1,000,000) was applied to the datasets. Since the outcome variable was binary, we expected logistic regression to be applied. However, DHS data has a hierarchical nature which violates the assumption of independence of observations since women within similar clusters are more likely to be related than women in another cluster. Thus, a multilevel regression model as an analysis tool is expected by nature. Hence, a multilevel logistic regression was employed to identify associated factors.

#### Model building process

This study had binary outcomes: whether a woman with a live birth 5 years preceding the surveys received two or more tetanus toxoid injections during pregnancy for her most recent birth. The model-building process began with a null model (model I), and complex models such as a model containing individual and household-level factors (model II), a model containing only community-level factors (model III), and a model with individual, household, and community-level factors (model IV) were built step by step. We compared these models using log likelihood (LL) and information criteria (AIC and BIC). Model IV was a better-fitting model. Then a bi-variable and multivariable two-level logistic regression model was fitted to identify determinant factors of adequate TT vaccination. The variability in the odds of TT immunization explained by successive models was estimated by proportional change in variance (PCV) as follows:

$$\mathrm{PCV}=\frac{\mathrm{VA}-\mathrm{VB}}{\mathrm{VA}}\;*\;100$$

where VA and VB are the neighborhood variance in the empty model and the variance in the successive models (40).

#### Parameter estimation

##### The fixed effects

It represents the observed quantities or it is a measure of association used to assess the relationship between likelihood of adequate TT vaccination and independent variables at individual and community levels. The relationship between adequate TT vaccination and other variables was determined by multivariable analysis of the selected model and an adjusted OR with a 95% confidence interval (CI) and *p*-value ≤ 0.05 were used to declare significant factors.

##### The random effect

The intra-class correlation coefficient (ICC), and median odds ratio (MOR) were used to quantify the unexplained heterogeneity of the outcome across areas. The MOR is the median value of the OR between the highest and lowest risk areas when randomly picking out two areas (40). The ICC can summarize the proportion of the total variance accounted for by higher-level units (41).

$$\mathrm{ICC}=\frac{\sigma_{u}^{2}}{\sigma_{u}^{2}+\sigma_{e}^{2}}$$

$$\mathrm{MOR}=\exp\left[\sqrt{\left(2\times V_{A}\right)}\times 0.6745\right]=\exp\left(0.95\left(\sqrt{V_{A}}\right)\right)$$

where *V_A_* is the area level variance, and 0.6745 is the 75th centile of the cumulative distribution function of the normal distribution with mean 0 and variance 1 (40).

#### Ethical considerations

The study was a secondary data analysis based on the publicly available DHS datasets; thus, ethical approval and participant consent were not necessary. Nevertheless, we asked the MEASURE DHS Program for the data, and we were permitted to download and utilize it.

## Results

### Characteristics of the study population

A total of 84,248 women with a live birth 5 years preceding the surveys and two or more tetanus toxoid injections received during the pregnancy of the most recent birth were included. More than one-fourth (26.01%) were from Nigeria, and nearly half (47.1%) were between the age brackets of 25 and 34 years. Almost all (94.24%) were married. Regarding employment status, about two-thirds (66.49% and 65.98%) were unemployed and had no media exposure, respectively. The majority (83.94%) of the participants had given birth before the age of 24 years. More than half (53.25%) had no history of health facility visits within 12 months before the surveys. About 39.33% had at least 4 ANC visits. More than three-fourth (77.73%) had not received postnatal examinations for their children. More than two-thirds (67.9%) of the participants lived in rural areas (Table 2).

**Table 2:** Characteristics of the study population.

Variables	Categories	Weighted frequency	Percentage (%)
Country	Afghanistan	19,633	23.30
	Angola	8,495	10.08
	Nigeria	21,911	26.01
	Papua New Guinea	6,759	8.02
	Pakistan	6,711	7.97
	Yemen	20,739	24.62
Maternal age	15–24	23,374	27.74
	25–34	39,684	47.10
	35+	21,190	25.15
Marital status	Single	2,226	2.64
	Married	79,395	94.24
	Widowed	828	0.98
	Divorced	580	0.69
	Separated	1,219	1.45
Mother occupation	Working	28,232	33.51
	Not working	56,016	66.49
Mass media exposure	No	28,660	34.02
	Yes	55,588	65.98
Age at first birth	<24	70,714	83.94
	24–30	11,914	14.14
	>30	1,620	1.92
Parity	Primipara	15,066	17.88
	Multipara	39,572	46.97
	Grand multipara	29,610	35.15
Decision on respondent's health care	Respondent alone	8,131	9.65
	Joint decision	32,761	38.89
	Husband alone	35,056	41.61
	Other	8,300	9.85
Sex of household head	Male	76,491	90.79
	Female	7,757	9.21
Health visits	No	39,388	46.75
	Yes	44,860	53.25
ANC visit	No	24,624	29.23
	1–3	25,059	29.74
	4 and more	33,138	39.33
	Don't know	1,427	1.69
Iron supplementation	No	38,614	45.83
	Yes	44,519	52.84
	Don't know	1,115	1.32
Ever had terminated pregnancy	No	68,473	81.28
	Yes	15,775	18.72
Post-natal check up	No	65,488	77.73
	Yes	18,760	22.27
Household level factors			
Wealth index	Poorest	17,359	20.60
	Poorer	17,613	20.91
	Middle	17,214	20.43
	Richer	16,604	19.71
	Richest	15,458	18.35
Health insurance	No	82,502	97.93
	Yes	1,746	2.07
Community level factors			
Residence	Urban	27,883	33.10
	Rural	56,365	66.90
Distance to health facility	Big problem	43,751	51.93
	Not big problem	40,497	48.07
Place of delivery	Home and other	45,771	54.33
	Health facility	38,477	45.67
Community level media exposure	Low	53,949	64.04
	High	30,299	35.96
Community level poverty	Low	30,196	35.84
	High	54,052	64.16

### Prevalence of adequate TT imunization among pregnant women

**Figure 1:**
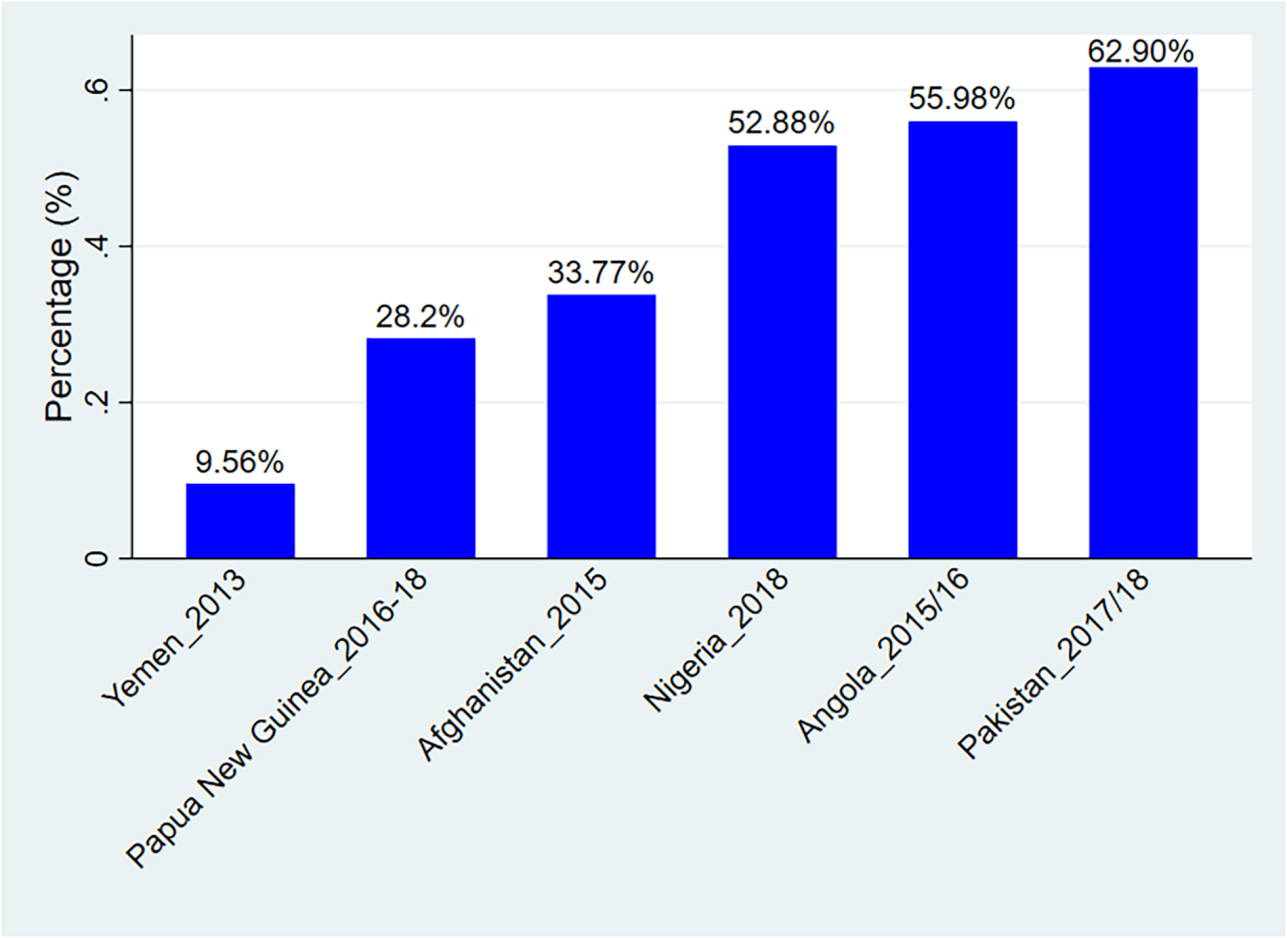
Prevalence of adequate TT injections uptake among women in countries that didn’t eliminate tetanus by 2024.

The prevalence of adequate TT vaccine during pregnancy among women living in countries that haven't eliminated tetanus by 2024 was 36.89% [95% CI: 36.57–37.22%], with the highest proportion in Pakistan (62.90%) and the lowest proportion in Yemen (9.56%) Figure 1.

### Factors associated with adequate TT injections during pregnancy

#### Random effect analysis

In the null model, the ICC value of 17.78% [95% CI: 16.25, 19.41%] indicated that about 17.78% of the overall variability in adequate TT injections was explained by the between-cluster variation. The MOR was also 2.24 [95% credible interval: 2.14, 2.33]. It was greater than one, and the 95% credible interval clearly excluded the value of 1, which indicates there were strong area-level differences in the probability of having adequate TT injections during pregnancy. If we randomly select two pregnant women from different clusters and transfer a woman from clusters with poor TT injection uptake to clusters with adequate TT injection uptake, the probability of having adequate TT injection uptake will (in median) increase by 2.24 times. The Likelihood Ratio test was also significant [LR test vs. logistic model: chibar2 (01) = 4061.52, prob ≥ chibar2 = 0.000], which showed that the mixed-effects models were the better-fitted model for this data compared to the classical model. With the final model, 85.92% of the variation was explained. This means that the predictor variables included in the final model accounted for 85.92% of the variation in adequate TT vaccination. Candidate models were compared using log likelihood (LL), AIC, and deviance. Thus, a model with all individual, household, and community-level factors (Model-IV) was selected as a better-fitted model (Table 3).

**Table 3:** Comparison of model fitness measures among candidate multilevel models.

Model	Null	Model-I	Model-II	Model-III
LL	−54,499.99	−44,482.5	−51,773.14	−43,984.24
Degree of freedom	2	32	6	36
Deviance	1,08,999.98	88,965	1,03,546.28	87,968.48
AIC	1,09,004	89,029	1,03,558.3	88,040.48
BIC	1,09,022.7	89,328.52	1,03,614.5	88,377.38
ICC (95% CI)	17.78 (16.25, 19.41)	10.47 (9.38, 11.66)	12.29 (11.11, 13.57)	0.90 (0.08, 0.10)
MOR (95% CI)	2.24 (2.14, 2.33)	1.81 (1.74,1.87)	1.91 (1.84, 1.98)	1.39 (1.43, 1.46)
Variance	0.71	0.38	0.46	0.10
PCV	Base	46.48%	35.21%	85.92%

#### Fixed effect analysis

In our final model (Model IV), factors such as marital status, employment, household wealth index, age at first birth, parity, the person who decides on respondents health care, sex of the household head, history of health facility visits, number of ANC visits, iron supplementation, history of termination pregnancy, post-natal checkup, residence, distance to a health facility, place of delivery, community level media exposure, and community poverty were significantly associated with adequate TT uptake among pregnant women.

Compared to unmarried pregnant women, married women had a 36% [AOR = 1.36, 95% CI: 1.20, 1.54] increased odds of receiving enough TT injections. The odds of TT vaccine uptake was 32% lower [AOR = 0.68, 95% CI: 0.66, 0.71] among women not working during the survey as compared to their counter. The odds of TT vaccine uptake was 46% [AOR = 1.46, 95% CI: 1.36, 1.57], 48% [AOR = 1.48, 95% CI: 1.39, 1,59], 33% [AOR = 1.33, 95% CI: 1.26, 1.42], and 19% [AOR = 1.19, 95% CI: 1.13, 1.26] higher among pregnant women from the poorest, poorer, middle, and richer household wealth quintiles as compared to those from the richest wealth quintiles.

The odds of adequate TT vaccine was 10% [AOR = 1.10, 95% CI: 1.04, 1.16] higher among pregnant women who gave their first birth within the age bracket of 24–30, compared to those who gave their first birth before the age of 24. Primiparous women had a 9% higher odds [AOR = 1.09; 95% CI: 1.02, 1.17] of adequate TT vaccine during their last pregnancy compared to grand multiparous women. The odds of adequate TT injections was 13% [AOR = 1.13, 95% CI: 1.06, 1.20] and 49% higher [AOR = 1.49, 95% CI: 1.36, 1.65] among pregnant women whose healthcare is determined jointly and by someone other than a couples, respectively. The odds of adequate TT vaccine uptake was 13% higher [AOR = 1.13; 95% CI: 1.06, 1.20] when the household head was female. Women who had not visited health facilities within 12 months prior to the survey had a 28% lower odds [AOR = 0.72, 95% CI: 0.70, 0.75] of adequate TT injections during their last pregnancy.

Pregnant women with an ANC visit number of 1–3, 4+ ANC visits, and who don't know the number of ANC visits had a 3.08 [AOR = 3.08, 95% CI: 2.91, 3.27], 5.94 [AOR = 5.94, 95% CI: 5.60, 6.30], and 3.44 [AOR = 3.44, 95% CI: 3.03, 3.91] times higher odds of adequate TT injections. The odds of adequate TT vaccination was 2.43 [AOR = 2.43, 95% CI: 2.34, 2.53] and 1.80 [AOR = 1.80, 95% CI: 1.54, 2.10] times higher among women who received iron supplementation and who didn't remember as compared to who didn't take iron supplementation, respectively. The odds of TT injections in women who had terminated pregnancy was 11% lower [AOR = 0.89, 95% CI: 0.85, 0.93] than their counterparts. The odds of adequate TT injections in postnatal women increased by 18% [AOR = 1.18, 95% CI: 1.13, 1.23] when compared to women who did not. Women who were living in rural areas had a 20% decreased [AOR = 0.80, 95% CI: 0.77, 0.84] odds of adequate TT injections compared to those living in urban areas. Women who had no problem with distance to health facilities had a 22% increased [AOR = 1.22, 95% CI: 1.18, 1.27] odds of adequate TT injections compared to their counter. Giving birth at a health facility had a 41% [AOR = 1.17, 95% CI: 1.36, 1.47] increased odds of adequate TT vaccination compared to those who gave birth at home or other places. Women from clusters with low community media exposure and high community-level poverty had a 26% lower [AOR = 0.74, 95% CI: 0.67, 0.81] odds of adequate TT injections during pregnancy, respectively (Table 4).

**Table 4:** Results of Bi-variable and multivariable multilevel logistic regression of adequate TT vaccination during pregnancy among 6 countries not achieved MNTE.

Variables	COR (95% CI)	AOR (95% CI)			
		Null (model I)	Model II	Model III	Model IV
Maternal age					
15–24	1.00	—	1.00	—	1.00
25–34	0.97 (0.93, 1.00)	—	1.03 (0.98, 1.08)	—	1.02 (0.97, 1.07)
35+	0.85 (0.82, 0.89)**	—	1.00 (0.93, 1.07)	—	0.98 (0.92, 1.05)
Marital status					
Single	1.00	—	1.00	—	1.00
Married	0.56 (0.51, 0.61)**	—	1.33 (1.17, 1.50)**	—	1.36 (1.20, 1.54)**
Widowed	0.79 (0.66, 0.93)**	—	1.18 (0.97, 1.43)	—	1.20 (0.99, 1.45)
Divorced	0.68 (0.56, 0.83)**	—	0.93 (0.75, 1.16)	—	0.93 (0.74, 1.15)
Separated	0.89 (0.77, 1.04)	—	0.97 (0.83, 1.13)	—	0.96 (0.82, 1.12)
Mother employment					
Working	1.00	—	1.00	—	1.00
Not working	0.46 (0.44, 0.47)**	—	0.69 (0.66, 0.71)**	—	0.68 (0.66, 0.71)**
Mass media exposure					
No	1.00	—	1.00	—	1.00
Yes	1.56 (1.51, 1.62)**	—	1.06 (1.02, 1.11)**	—	1.01 (0.97, 1.05)
Household wealth index					
Poorest	0.40 (0.38, 0.42)**	—	1.11 (1.03, 1.18)**	—	1.46 (1.36, 1.57)**
Poorer	0.53 (0.50, 0.56)**	—	1.17 (1.10, 1.24)**	—	1.48 (1.39, 1,59)**
Middle	0.68 (0.65, 0.72)**	—	1.13 (1.07, 1.20)**	—	1.33 (1.26, 1.42)**
Richer	0.85 (0.80, 0.89)**	—	1.09 (1.03, 1.15)**	—	1.19 (1.13, 1.26)**
Richest	1.00	—	1.00	—	1.00
Age at first birth					
<24	1.00	—	1.00	—	1.00
24–30	1.30 (1.24, 1.35)**	—	1.12 (1.06, 1.18)**	—	1.10 (1.04, 1.16)**
>30	1.39 (1.24, 1.54)**	—	1.16 (1.02, 1.32)*	—	1.12 (0.99, 1.12)
Parity					
Primipara	1.53 (1.47, 1.60)**	—	1.14 (1.07, 1.22)**	—	1.09 (1.02, 1.17)*
Multipara	1.24 (1.20, 1.29)**	—	1.05 (1.01, 1.10)*	—	1.03 (0.98, 1.08)
Grand multipara	1.00	—	1.00	—	1.00
Decision on respondent's health care					
Women alone	1.00	—	1.00	—	1.00
Joint decision	0.93 (0.89, 0.99)**	—	1.10 (1.03, 1.17)	—	1.13 (1.06, 1.20)**
Husband alone	0.75 (0.71, 0.79)**	—	1.05 (0.99, 1.12)	—	1.06 (0.99, 1.13)
Other	1.23 (1.15, 1.32)	—	1.47 (1.34, 1.63)	—	1.49 (1.36, 1.65)**
Sex of household head					
Male	1.00	—	1.00	—	1.00
Female	1.75 (1.67, 1.84)**	—	1.16 (1.10, 1.23)	—	1.13 (1.06, 1.20)**
Health visits					
No	0.51 (0.50, 0.53)**	—	0.71 (0.69, 0.74)	—	0.72 (0.70, 0.75)**
Yes	1.00	—	1.00	—	1.00
ANC visit					
No	1.00	—	1.00	—	1.00
1–3	5.37 (5.11, 5.65)**	—	3.19 (3.01, 3.37)**	—	3.08 (2.91, 3.27)**
4 and more	14.2 (13.52,14.92)**	—	6.43 (6.07, 6.81)**	—	5.94 (5.60, 6.30)**
Don't know	7.66 (6.81, 8.61)**	—	3.74 (3.30, 4.24)**	—	3.44 (3.03, 3.91)**
Iron supplementation					
No	1.00	—	1.00	—	1.00
Yes	6.15 (5.93, 6.38)**	—	2.49 (2.39, 2.59)**	—	2.43 (2.34, 2.53)**
Don't know	4.04 (3.54, 4.61)**	—	3.18 (2.76, 3.66)**	—	1.80 (1.54, 2.10)**
Ever had terminated pregnancy					
No	1.00	—	1.00	—	1.00
Yes	0.89 (0.85, 0.92)**	—	0.90 (0.86, 0.94)**	—	0.89 (0.85, 0.93)**
Health insurance					
No	1.00	—	1.00	—	1.00
Yes	1.50 (1.35, 1.67)**	—	1.02 (0.91, 1.13)	—	1.00 (0.90, 1.12)
Post-natal check up					
No	1.00	—	1.00	—	1.00
Yes	1.79 (1.72, 1.85)**	—	1.19 (1.15, 1.24)	—	1.18 (1.13, 1.23)**
Distance to health facility					
Big problem	1.00	—	1.00	—	1.00
Not big problem	1.86 (1.80 1.92)**	—	1.25 (1.21, 1.30)	—	1.22 (1.18, 1.27)**
Community level factors					
Residence					
Urban	1.00	—	—	1.00	1.00
Rural	0.46 (0.44, 0.48)**	—	—	0.59 (0.57, 0.61)**	0.80 (0.77, 0.84)**
Place of delivery					
Home and other	1.00	—	—	1.00	1.00
Health facility	2.70 (2.62, 2.80)**	—	—	2.40 (2.33, 2.48)**	1.41 (1.36, 1.47)**
Community level media exposure					
Low	0.44 (0.39, 0.48)**	—	—	0.65 (0.59, 0.71)**	0.74 (0.67, 0.81)**
High	1.00	—	—	1.00	1.00
Community level poverty					
Low	1.00	—	—	1.00	1.00
High	0.46 (0.41, 0.51)**	—	—	0.87 (0.79, 0.96)**	0.93 (0.85, 1.02)

* *p* < 0.05.

** *p* < 0.01.

## Discussion

The current study examined the relationship between several demographic, socioeconomic, and health-care access-related factors and adequate TT immunization among women from countries that have not achieved MNTE and gave birth within the previous 5 years. The pooled prevalence of adequate TT injections during the most recent pregnancy was 40.89% (95% CI: 25.49–56.28%). Previous findings from Brazil (30), Ethiopia (25, 31, 32, 35), Sudan (26), Bangladesh (5, 42), Indonesia (43), eastern Africa (21), and Benin (44) are lower than ours. This discrepancy could be brought about by a difference in the coverage of TT vaccines (45). Moreover, misreporting of vaccination rates and conspiracy theories regarding the vaccine could be factors in the disparity (14).

Compared to single women, married women had a higher odds of adequate TT vaccination. This finding was supported by evidence from Cameroon (33). This can be explained by the fact that single mothers, especially those who have unwanted pregnancies, may not have social and financial support, so they may give little attention to themselves and their baby (33). In addition, married women have a higher probability of attending and completing ANC visits (21–23, 26, 27, 46, 47). Our analysis also revealed that pregnant women who received ANC visits had a higher odds of adequate TT vaccination, which lends further support to this finding. This might be due to the positive effect of health education and advice they received about the importance of TT immunization from health professionals. That might be the reason that the prevalence of adequate TT injection was higher among women whose health care is decided by others compared to respondents alone in our study. A possible justification may be that women may develop a positive attitude when they are told about health service utilization by their colleagues or other people who have knowledge about maternal and child health.

The odds of adequate TT vaccination were lower for women without employment. This finding is consistent with studies conducted in Kenya (48), Bangladesh (42), Ethiopia (27), and sub-Saharan Africa (29). A possible explanation for our finding might be that working women could have more social support, communication, and information exchange regarding maternal and child service at their workplace compared to non-working women's behavior (27, 49). Thus, non-working women might loss the opportunities for learning and engagement in health-related dialogues, particularly during pregnancy, and their health service utilization behavior.

Giving birth at an older age increased the odds of an adequate TT vaccine compared to those who gave birth before the age of 24. Previous findings support our result (21, 34). This could be due to the positive effect of increased age on improved attitudes and knowledge (50). This may be related to the decision-making power of women. Empowered women had a right to decide on the appropriate age of birth and health care service utilization, which can significantly influence fertility and reproductive health-related issues (51). In our study, the odds of adequate TT vaccine uptake was higher among pregnant women when the household head was female. This shows that when women become the head of the house, they become more empowered to decide on ANC visits and other maternal health care services (52). Furthermore, our study indicated women who had not visited health facilities within 12 months prior to the survey had a lower odds of adequate TT injection. This might also be related to women's decision-making power. Individuals with empowerment will be able to get freely (28, 51).

The odds of adequate TT vaccine uptake during pregnancy was higher among primiparous women compared with grand multiparous. However, studies from Ethiopia (27, 35, 50, 53) found no association between parity and TT immunization. A possible explanation might be that multiparous women have experience with pregnancy and delivery. Another finding of this study revealed that women who received iron supplementation during their pregnancy had higher odds of adequate TT vaccination compared to those who didn't take iron supplementation. This might be a clear indication that pregnancy experience is vital to pregnancy-related health service utilization because women who received iron supplements may also receive information about the importance of TT immunization. A previous study also supported this finding (27). This might be because iron supplements are common and recommended services during pregnancy (54).

Compared to their counterparts, women with a history of abortion had a reduced odds of receiving adequate TT injections. However, this finding is in contrast to a study conducted among east African women (21), where termination of pregnancy had no association with tetanus immunization. This might be because of cultural, behavioral, and health care system differences across countries included in the studies, since our study included women from different continents. Our finding might also indicate the contribution of poor maternal health service utilization to abortion.

Having a postnatal examination increased the odds of adequate TT injections compared to their counterparts. Previous findings from Cameroon supported this finding (33). When a pregnant woman goes to a health facility to take TT vaccination and attends an ANC visit, the health worker emphasizes the importance of timely infant vaccination and communicates the infant/child vaccination schedule (55). Thus, women who have received the TT vaccination may have better knowledge about the importance of postnatal checkups. Thus, they are more likely to be vaccinated.

Compared to urban residents, the odds of sufficient TT injections was lower among women living in rural areas. This result is in line with research from Ethiopia (31, 35) and Bangladesh (5). This could be due to the disparity in the use of maternal healthcare services between rural and urban locations, which is influenced by different socioeconomic conditions (56). The quality of health services, accessibility, and information provision are better in urban areas. Furthermore, our research showed that women without an issue with distance to health facilities had a higher odds of adequate TT injections. Rural residents could find it difficult to access health facilities. This result is in line with earlier studies (21, 25, 28, 31, 36). Women residing in remote healthcare facilities might not have enough time to travel to a healthcare facility or enough money to cover the cost of transportation (21).

Women who gave birth in a health facilities had a higher odds of adequate TT immunization than those who gave birth at home or somewhere else. Our findings have been supported by other research (5, 21). This may be connected to the information that the medical staff provided during the ANC visit, meaning that women who were eligible for the TT vaccination also had the opportunity to learn about the significance of giving birth in a medical facility. Pregnant women from clusters with limited exposure to community media were less likely to receive appropriate TT injections. Our conclusion is corroborated by earlier studies (27). Our results could be explained by the possibility that women who are adequately informed about the tetanus vaccine will be more likely to accept vaccination during pregnancy (57). Thus, the media plays a vital role in delivering this information to women.

## Strength and limitations of the study

By utilizing the most recent data sets from nations that have not attained MNTE, our study has contributed a lot to the understanding of factors related to adequate TT vaccination. To identify variables associated with adequate TT vaccination, we used a robust analysis technique. But this analysis made use of DHSs from six nations, each carried out in a separate year. This may have population and economic disparities that this study was unable to address. The information gathered from the surveys provided is cross-sectional. Therefore, we can't establish causality using a cross-sectional study.

## Conclusion

Marital status, wealth index, age at first birth, parity, the person who makes health care decisions for the respondent, the sex of the household head, maternal employment status, ANC visits, postnatal checkups, health facility visits, history of abortion, health insurance, distance to a health facility, residence, and community media exposure were all linked to adequate TT vaccination during pregnancy. In order to improve maternal and neonatal health, our research offered helpful recommendations for personal, domestic, and community-based prevention and interventions. The level of sufficient tetanus toxoid immunization in nations that have not yet reached MNTE will therefore be improved by promoting maternal employment, increasing post-abortion care, improving health accessibility, encouraging pregnant women to receive ANC and postnatal care, emphasizing the benefits of TT immunization during these services, increasing media coverage and community literacy, and further bolstering free maternal health services.

## Data Availability

Publicly available datasets were analyzed in this study. This data can be found here: http://www.dhsprogram.com.
